# Enhanced Recovery After Surgery (ERAS) Approach: A Medical Complex Experience

**DOI:** 10.7759/cureus.51208

**Published:** 2023-12-28

**Authors:** Abdelfatah M Elsenosy, Eslam Hassan, Mujtaba Abdelgader, Omar S Elgamily, Abdelhares Hegazy

**Affiliations:** 1 Trauma and Orthopaedics, University Hospitals Dorset, Poole, GBR; 2 Trauma and Orthopaedics, Poole General Hospital, Poole, GBR; 3 General Surgery, Cairo University, Cairo , EGY; 4 General Surgery and Surgical Oncology, Maadi Armed Forces Medical Complex, Cairo, EGY

**Keywords:** outcomes, eras, colorectal, recovery, surgery

## Abstract

Background: Enhanced recovery after surgery (ERAS) is a multimodal, multidisciplinary approach aimed at reducing organ failure and mitigating stress reactions in surgery patients. This investigation sought to assess available data concerning the benefits of ERAS protocols in improving patient outcomes for individuals undergoing significant colorectal surgery.

Methods: The study involved 65 patients who underwent colectomy and lower anterior resection for rectal cancers. Patients were divided into three groups: Group 1 comprised 22 patients enrolled retrospectively who received the traditional protocol; Group 2 consisted of 20 patients enrolled prospectively who received the ERAS protocol; and Group 3 included 23 patients enrolled retrospectively who received the ERAS protocol. Each patient underwent a comprehensive history, physical examination, laboratory testing, computed tomography, MRI, and chest radiography.

Results: Hospital stay durations were significantly shorter in both ERAS groups during the first and second cycles compared to the non-ERAS group (P<0.001, <0.001), with no significant difference between ERAS groups in either cycle. Delayed intestinal motility was significantly more pronounced in the non-ERAS group compared to ERAS groups in both cycles (P=0.005), with only five (22.7%) cases reported in the non-ERAS group.

Conclusion: ERAS implementation in the perioperative management of colorectal surgery patients is associated with improved outcomes and shorter recovery times. Implementation of ERAS in hospitals is feasible and beneficial.

## Introduction

The goal of enhanced recovery after surgery (ERAS) is to expedite full recovery postsurgery by minimizing stress response and organ dysfunction [1]. ERAS comprises three main components: preoperative, intraoperative, and postoperative care. Preoperatively, it involves counseling, anesthesiology consultation, and, for major surgeries, carbohydrate loading [2]. The intraoperative phase includes strategies to prevent postoperative nausea and vomiting, multimodal pain relief, maintaining normal body temperature, monitoring, and hydration [3]. Postoperatively, ERAS focuses on adequate fluid therapy, preventing postoperative ileus, glycemic control, urinary drainage, nutritional support, improving cardiopulmonary function, prompt restoration of bowel function, and early mobilization [4].

ERAS benefits patients undergoing colorectal surgery by reducing postoperative complications, enhancing healing, and shortening hospital stays. While extensively adopted in Europe with positive outcomes, implementing ERAS programs in regions with fragmented healthcare systems poses challenges [5]. The applicability and benefits of ERAS in urgent colorectal surgeries remain less understood. Challenges in studying emergency colorectal diseases might be linked to the absence of specific ERAS guidelines for such cases [6,7].

This investigation aims to assess existing data on the advantages of ERAS protocols in improving outcomes for individuals undergoing major colorectal surgery.

## Materials and methods

This study involved 65 patients who had colectomy and lower anterior resection for rectal cancers at the General Surgery & Surgical Oncology Department in Maadi Armed Forces Medical Complex. The research was conducted after approval from the ethics committee. Data were collected from patient records between August 1, 2020, and December 15, 2020. After digitalizing ward notes, we collected data prospectively from January 2021 to May 2021. We implemented changes on postoperative day 1 (POD 1) that included removing the nasogastric (NG) tube, encouraging patients to walk, removing catheters, and starting oral feeding. These changes were applied and data were collected prospectively. Patient identification included name, birthdate, hospital, and address identification number. There were 42 patients in the first cycle and 23 in the second. The surgeries were performed using lap-assisted procedures. Patients with blockages, peritonitis, or distant metastases were not included in the trial. The audit cycle is as follows: Problem: Prolonged hospital stays causing stress, weight loss, and increased use of hospital resources. The standard used is ERAS protocol for colorectal patients.

Data collection and analysis

Twenty-two patients were enrolled retrospectively, and 20 were enrolled prospectively, all undergoing the ERAS protocol. In the second cycle, we collected data from 23 patients retrospectively.

Implementation

Changes were made concerning the NG tube, oral feeding, urinary catheter, and postoperative mobility.

Reaudit

Retrospective data collection ensured the sustained application of changes.

Grouping

Patients were divided into three groups: Group 1: 22 patients with traditional protocol (retrospective), Group 2: 20 patients with ERAS protocol (prospective), and Group 3: 23 patients with ERAS protocol (retrospective).

Assessments

Patients underwent thorough medical history reviews, comprehensive laboratory tests including tumor markers, MRI, CT scans, chest radiography, and abdominal ultrasound.

Interventions

ERAS protocols varied but commonly involved preoperative counseling, carbohydrate loading, avoiding prolonged fasting, early mobilization, early catheter removal, and multimodal pain relief. A multidisciplinary team including surgeons, anesthesiologists, nurses, and others implemented these protocols.

Preoperative care

Before the surgery, the patient received detailed information about the procedure. For certain surgeries like right side resection or colostomy reversal, no enemas were needed. However, for lesions on the left side and in the rectal area, one enema was given at night and another in the morning. Four hours prior to surgery, the patient had clear fluids high in carbohydrates (CHO). Metronidazole and cefoperazone were given through IV 12 hours and 30 minutes before surgery. Subcutaneous clexane was given the night of the procedure.

Intraoperative care

During surgery, NG tubes were sometimes used and removed early. Hemodynamic and central venous pressure (CVP) monitoring was used to manage warm IV fluids. Epidural analgesia and epidural bupivacaine infusion were used in some cases. Drains were infrequently used, and sometimes only one was used.

Postoperative care

After surgery, the patient's diet gradually progressed: sipping water and gums on the evening of the operation, clear fluids the next day, and semi-solid foods on the second day if tolerated. If a catheter was used, it was removed on the same day as the surgery. Early mobilization started by sitting on the bed and then leaving it on the day of the surgery. The patient spent two hours out of bed on the first day after surgery and six hours on the second day. Drains were removed the day after surgery if they contained a small amount of fluid and if no abdominal accumulation was found. IV fluids were stopped on the second day. Intravenous paracetamol (Perfalgan) was used for pain relief. Metoclopramide (Primperan) was given IV to prevent vomiting; if not effective, ondansetron (Zofran) was used.

Postoperative care and follow-up

Before discharge, the patient had to meet specific criteria: full mobility, oral pain medication, passing gas and stool, and tolerating solid foods without nausea or vomiting. Follow-up appointments were scheduled one week and one month after surgery.

Outcome measures

Primary outcomes included hospital stay length, survival rates, readmission and re-exploration rates, and postoperative complications (such as pulmonary thromboembolism, anastomotic leakage, and intestinal obstruction). Secondary measures involved pain levels measured using the visual analogue scale (VAS) [8], time to pass gas, first solid meal, and bowel function as assessed by PONV [9].

Statistical analysis

IBM SPSS Statistics for Windows, Version 28 (Released 2021; IBM Corp., Armonk, New York, United States) was used for statistical analysis. The ANOVA (F) test compared quantitative variables, presented as mean and standard deviation (SD). The Chi-square test analyzed frequency and percentage (%) of qualitative variables. A P value less than 0.05 was considered statistically significant.

## Results

Concerning the baseline characteristics, age was significantly higher in ERAS in the second cycle group compared to no ERAS and ERAS in the first cycle groups (P<0.001, <0.001) and was significantly greater in ERAS in the first cycle group in contrast to the no ERAS group (P<0.001). Sex, BMI, ASA, smoking, comorbidities (HTN, DM, hepatic, COPD, and stroke), and the type of operation done were insignificantly different among the studied groups (Table 1).

**Table 1:** Baseline characteristics of the studied patients Data are presented as mean ± SD or frequency (%), ERAS: enhanced recovery after surgery, BMI: body mass index, ASA: American Society of Anesthesiologists,  HTN: hypertension, DM: diabetes mellitus, COPD: chronic obstructive pulmonary disease, LAR: lower anterior resection, RT: right, APR: abdominoperineal resection, *: statistically significant as P value <0.05, P1: p value between no ERAS and ERAS in the first cycle, P2: p value between no ERAS and ERAS in the second cycle, P3: P1: p value between ERAS in the first cycle and the second cycle.

		No ERAS (n=22)	ERAS in the first cycle (n=20)	ERAS in the second cycle (n=23)	P value	Post hoc
Age (years)		46.05 ± 3.73	60.55 ± 3.14	72.87 ± 4.94	<0.001*	P1 <0.001*
						P2 <0.001*
						P3 <0.001*
Sex	Male	9 (40.9%)	14 (70%)	13 (56.5%)	0.165	---
	Female	13 (59.1%)	6 (30%)	10 (43.5%)		
BMI (Kg/m^2^)		26.18 ± 4.39	25.15 ± 4.16	27.65 ± 3.7	0.138	---
ASA	ASA I	2 (9.1%)	2 (10%)	2 (8.7%)	0.826	
	ASA II	14 (63.6%)	14 (70%)	18 (78.3%)		
	ASA III	6 (27.3%)	4 (20%)	3 (13%)		
Smoking		5 (22.7%)	4 (20%)	6 (26.1%)	0.893	---
Comorbidities	HTN	2 (9.1%)	3 (15%)	2 (8.7%)	0.763	---
	DM	3 (13.6%)	1 (5%)	2 (8.7%)	0.623	
	Hepatic	2 (9.1%)	1 (5%)	1 (4.3%)	0.777	---
	COPD	0 (0%)	0 (0%)	0 (0%)	---	---
	Stroke	1 (4.5%)	0 (0%)	1 (4.3%)	0.631	
Operation done						
LAR		4 (18.2%)	4 (20%)	7 (30.4%)	0.815	---
Extended RT hemicolectomy		5 (22.7%)	6 (30%)	8 (34.8%)		
Subtotal colectomy		4 (18.2%)	2 (10%)	4 (17.4%)		
APR		7 (31.8%)	6 (30%)	3 (13%)		
Sigmoidectomy		2 (9.1%)	2 (10%)	1 (4.3%)		

Preoperative measures

Before surgery, there were significant differences among the groups (P<0.05). In the no ERAS group, all patients received counseling and were prepared through frequent enemas and lactulose. Preoperative use of anticoagulants was notably higher in both ERAS groups in the first and second cycles compared to the no ERAS group (P<0.001) (Table 2).

**Table 2:** Perioperative measures of the studied patients Data are displayed as mean ± SD or frequency (%), ERAS: enhanced recovery after surgery, CHO: carbohydrate, IVF: intravenous fluid, *: statistically significant as P value<0.05, P1: p value between no ERAS and ERAS in the first cycle, P2: p value between no ERAS and ERAS in the second cycle, P3: P1: p value between ERAS in the first cycle and the second cycle.

	No ERAS (n=22)	ERAS in the first cycle (n=20)	ERAS in the second cycle (n=23)	P value	Post hoc
Preoperative					
Patient counselling	19 (86.4%)	5 (25%)	7 (30.4%)	<0.001*	---
CHO load	0 (0%)	8 (40%)	5 (21.7%)	0.009*	---
Enema	14 (63.6%)	2 (10%)	3 (13%)	<0.001*	---
Lactulose	22 (100%)	3 (15%)	5 (21.7%)	<0.001*	---
Anticoagulant	4 (18.2%)	20 (100%)	20 (87%)	<0.001*	---
Intraoperative					
Nasogastric tube	22 (100%)	15 (75%)	17 (73.9%)	0.033*	---
Drain	22 (100%)	3 (15%)	4 (17.4%)	<0.001*	---
Transverse incision	0 (0%)	1 (5%)	2 (8.7%)	0.378	---
Hypothermia prevention	0 (0%)	2 (10%)	4 (17.4%)	0.130	---
Epidural analgesia	1 (4.5%)	4 (20%)	6 (26.1%)	0.141	---
Postoperative					
Day of first diet	6.55 ± 1.9	0.7 ± 0.73	1.09 ± 1.12	<0.001*	P1 <0.001*
					P2 <0.001*
					P3= 0.196
Day of mobilization	1.55 ± 0.34	0.4 ± 0.5	1.17 ± 1.19	<0.001*	P1 <0.001*
					P2= 0.167
					P3= 0.010*
IVF duration (days)	6.32 ± 0.78	2.1 ± 0.79	2.48 ± 0.9	<0.001*	P1 <0.001*
					P2 <0.001*
					P3= 0.153
Catheter duration (days)	1.55 ± 0.34	0.70 ± 0.47	0.65 ± 0.57	<0.001*	P1 <0.001*
					P2 <0.001*
					P3= 0.768

Intraoperative measures

During surgery, the use of nasogastric tubes and drains showed significant differences among the groups (P<0.001, <0.001). Every patient in the no ERAS group had nasogastric tubes and drains. However, factors like transverse incision, preventing hypothermia, and using epidural analgesia didn't show significant differences among the groups (Table 2).

Postoperative measures

After surgery, the time to start the first diet was notably earlier in both ERAS groups in the first and second cycles compared to the no ERAS group (P<0.001, <0.001). There were no significant differences between the ERAS groups in either cycle. The day of mobilization was earlier in the ERAS group in the first cycle compared to the no ERAS group and in the ERAS group in the second cycle (P<0.001, 0.010), with no significant difference between the no ERAS group and the ERAS group in the second cycle. Both IV fluid duration and catheter duration were significantly shorter in both ERAS groups in the first and second cycles compared to the no ERAS group (P<0.05). However, there were no significant differences between the ERAS groups in either cycle (Table 2).

The pain control methods varied significantly among the studied groups (P<0.001). According to the VAS pain scores, both ERAS groups in the first and second cycles had notably lower scores compared to the no ERAS group (P<0.001, <0.001). Interestingly, there were no significant differences between the ERAS groups in either cycle. Additionally, the duration of narcotic use was significantly shorter in both ERAS groups during both cycles compared to the no ERAS group (P<0.001, <0.001), but there were no notable differences between the ERAS groups within each cycle (Table 3).

**Table 3:** Postoperative pain control, pain score, and duration of narcotic use of the studied patients Data are displayed as mean ± SD or frequency (%), ERAS: enhanced recovery after surgery, NSAIDs: nonsteroidal anti-inflammatory drugs, VAS: visual analogue scale, *: statistically significant as P value<0.05, P1: p value between no ERAS and ERAS in the first cycle, P2: p value between no ERAS and ERAS in the second cycle, P3: P1: p value between ERAS in the first cycle and the second cycle.

		No ERAS (n=22)	ERAS in the first cycle (n=20)	ERAS in the second cycle (n=23)	P value	Post hoc
Pain control modality	Paracetamol	2 (9.1%)	15 (75%)	13 (56.5%)	<0.001*	---
	NSAIDS	13 (59.1%)	3 (15%)	6 (26.1%)		
	Opiates	5 (22.7%)	0 (0%)	0 (0%)		
	Epidural	2 (9.1%)	2 (10%)	4 (17.4%)		
Pain score (VAS)		6.68 ± 1.36	3.15 ± 0.81	3.7 ± 1.18	<0.001*	P1 <0.001*
						P2 <0.001*
						P3= 0.090
Duration of narcotic use (days)		6.68 ± 1.36	0.6 ± 0.5	0.87 ± 0.63	<0.001*	P1 <0.001*
						P2 <0.001*
						P3= 0.131

In terms of the primary outcome, the duration of hospital stays was notably shorter in both ERAS groups during the first and second cycles compared to the no ERAS group (P<0.001, <0.001). Interestingly, there were no significant differences between the ERAS groups in either cycle. Delayed intestinal motility was significantly more common in the no ERAS group compared to the ERAS groups in both the first and second cycles (P=0.005). Notably, this complication was reported in only five cases (22.7%) in the no ERAS group. Regarding other outcomes like readmission, wound infection, thromboembolism, and cardiac complications, there were no significant differences among the studied groups. Our study did not report cases of anastomotic leakage or mortality (Table 4).

**Table 4:** Primary outcome of the studied patients Data are displayed as mean ± SD or frequency (%), ERAS: enhanced recovery after surgery, *: statistically significant as P value<0.05, P1: p value between no ERAS and ERAS in the first cycle, P2: p value between no ERAS and ERAS in the second cycle, P3: P1: p value between ERAS in the first cycle and the second cycle.

	No ERAS (n=22)	ERAS in the first cycle (n=20)	ERAS in the second cycle (n=23)	P value	Post hoc
Hospital stay duration (days)	18.0 ± 4.4	6.0 ± 0.86	6.13 ± 0.81	<0.001*	P1 <0.001*
					P2 <0.001*
					P3= 0.612
Readmission	7 (31.8%)	1 (5%)	3 (13%)	0.057	---
Wound infection	2 (9.1%)	0 (0%)	0 (0%)	0.133	---
Anastomotic leakage	0 (0%)	0 (0%)	0 (0%)	---	---
Delayed intestinal motility	5 (22.7%)	0 (0%)	0 (0%)	0.005*	---
Thromboembolism	1 (4.5%)	0 (0%)	0 (0%)	0.370	---
Cardiac complications	2 (9.1%)	0 (0%)	0 (0%)	0.133	---
Mortality	0 (0%)	0 (0%)	0 (0%)	---	---

Regarding the secondary outcomes, the timing of the first flatus, first oral intake, and first solid meal were notably earlier in both ERAS groups during the first and second cycles compared to the no ERAS group (P<0.05). Interestingly, there were no significant differences between the ERAS groups in either cycle for these measures. Postoperative nausea and vomiting (PONV) occurred in four patients (18.2%) in the no ERAS group, one patient (5%) in the ERAS first cycle group, and three patients (13%) in the ERAS second cycle group, with no notable differences among the studied groups in terms of PONV incidence (Table 5).

**Table 5:** Secondary outcome of the studied patients Data are displayed as mean ± SD or frequency (%), ERAS: enhanced recovery after surgery, PONV: postoperative nausea and vomiting, *: statistically significant as P value<0.05, P1: p value between no ERAS and ERAS in the first cycle, P2: p value between no ERAS and ERAS in the second cycle, P3: P1: p value between ERAS in the first cycle and the second cycle.

	No ERAS (n=22)	ERAS in the first cycle (n=20)	ERAS in the second cycle (n=23)	P value	Post hoc
Time of first flatus (days)	5.09 ± 1.54	1.65 ± 0.49	1.48 ± 0.51	<0.001*	P1 <0.001*
					P2 <0.001*
					P3= 0.269
Time of first oral intake	4.5 ± 1.34 days	8.5 ± 2.33 h	8.52 ± 1.81 h	<0.001*	P1 <0.001*
					P2 <0.001*
					P3= 0.973
Time of first solid meal (days)	6.23 ± 0.92	3.0 ± 0.73	3.3 ± 0.97	<0.001*	P1 <0.001*
					P2 <0.001*
					P3= 0.258
PONV	4 (18.2%)	1 (5%)	3 (13%)	0.426	---

## Discussion

The use of ERAS procedures to enhance patient outcomes after major colorectal surgery has been assessed in a number of studies [10]. It was shown that, in comparison to traditional care, the use of ERAS protocols shortened the duration of hospital stay and had fewer postoperative problems. Furthermore, recommendations from the ERAS Society suggested using ERAS protocols to improve patient outcomes and speed up recovery following elective colon surgery [11].

Patients should be educated and expectations should be set, according to El-Shewy et al. program to guarantee well-established patient engagement [12]. Patients in the ERAS group were more prone to go through less pain, anxiety, and other side effects if they were told about the surgical technique, the postoperative course, to walk on the day of operation, and to return home in roughly three to five days. In order to promote stability and mobility following the procedure, this concentrated on strengthening the core and balance. This component was present in earlier research [13,14] and not included in others [15]. There has been a significant shift in the conventional settings away from extended fasting and toward allowing clear fluids, particularly CHO liquids, up to four hours before surgery in the ERAS group. Reduced catabolism, preserving nitrogen balance, enhanced insulin sensitivity after surgery, shorter hospital stays, and increased patient satisfaction due to decreased preoperative thirst, hunger, and discomfort are some advantages of the nutritious drink. With the exception of a few earlier trials, this component was present in the majority of the investigations that permitted CHO to load up to four hours before operation. Khoo et al. allowed fluid up to 3 h preoperatively. Teeuwen et al. [16] permitted fluid up to two hours prior to surgery [15]. For three days before to surgery, the non-ERAS group underwent numerous enemas, lactulose, and mannitol bowel preparation. In the ERAS group, El-Shewy et al. employed two enemas: one the night before surgery and one the morning of the operation for patients with rectosigmoid malignancy [12]. Khoo et al. utilized a standard bowel preparation [15]. Sarin et al. employed full bowel preparation for lesions on the left side and the rectal area, and no bowel preparation for resections on the right side [17].

Regarding the main findings, hospital stays were significantly shorter in both ERAS cycles compared to the no ERAS group, with no variation between ERAS cycles. There were no significant differences in wound infection, thromboembolism, or cardiac problems. Anastomotic leakage wasn't observed. In the secondary outcomes, both ERAS cycles showed earlier first flatus, oral intake, and solid meal times compared to the no ERAS group. Incidence of PONV varied across groups, with 4 (18.2%) in no ERAS, 1 (5%) in the first cycle, and 3 (13%) in the second cycle. Cochrane found ERPs reduced complications and LOS. The POWER study noted individual ERP components reduced complications and LOS, even outside established ERPs [18,19].

The more protocol components are applied, the better the patient results in centers with a well-established ERP. This relationship has been proposed between dosage effect and protocol adherence and patient outcomes. A multinational data collection spanning 13 centers at varying phases of ERAS adoption, involving over 2000 patients, demonstrated a negative correlation between adherence and the emergence of problems. 10. Among them are 13.1% for adherence below 50%, 11.6% for adherence between 75 and 90%, and 9.3% for adherence above 90% [20]. Gustafsson et al. showed that, as compared to institutions with less than 70% adherence, the incidence of five-year cancer-specific death was reduced by 42% in those with 70% or above adherence to ERAS components [11]. That being said, no evidence has been presented linking improved results to centers that follow an ERAS process but do not perform the items according to a predetermined routine.

Each and every perioperative session is impacted by ERP adherence. Certain recommendations, which can be regarded as standard care, have high adherence rates across all facilities. These include the laparoscopic approach, antibiotic and antithrombotic prophylaxis, and avoiding the use of nasogastric tubes. Nonetheless, certain items exhibit low adherence (< 50%) in both ERAS and non-ERAS contexts. After retrospectively analyzing 2876 patients in an ERP in colorectal surgery, Aarts et al. discovered that just 20.1% of the patients got care that satisfied every ERP phase [21]. Postoperative therapies that were independently linked to a higher incidence of optimum recovery had the lowest adherence rate (40.3%). This result is comparable with the adherence obtained in the POWER trial for these items and validates reports of low protocol adherence in the early postoperative phase [22]. In addition, Ripollés-Melchor et al. discovered that there was an independent relationship between two factors-early feeding and early mobilization, and a reduction in moderate-to-severe postoperative problems [19]. Early feeding (within 24 hours postoperatively) has been shown in previous research to speed gastrointestinal recovery and reduce the rate of complications and LOS.

Ripollés-Melchor et al. discovered that only in the highest quartile of ERP adherence (adherence >77%) was the average initial oral feeding administered less than 24 hours postoperatively [19]. This suggests that specialized centers are the only places where a direct intervention allowing the early start of oral feeding is carried out.

The meta-analysis conducted by Varadhan et al. demonstrated a significant decrease in the length of hospital stay, problems following surgery, and fatality rate related to the use of ERAS routes during major colorectal surgery [23]. These results are consistent with the objectives of ERAS protocols, which are to improve patient outcomes, reduce stress reactions following surgery, and promote recovery.

Several studies underscore the successful application of ERAS techniques. Simpson et al. showcased reduced complications, shorter hospital stays, and enhanced outcomes using ERAS in the UK [24]. Grass et al. also demonstrated benefits in elective colorectal surgery, emphasizing execution plans and interdisciplinary cooperation for desired results [25]. Adherence to ERAS guidelines is crucial for better outcomes, correlating higher compliance with fewer complications and shorter stays. Lee et al. showcased decreased hospital stays and increased infection rates with ERAS adoption in laparoscopic colon procedures at a metropolitan hospital [26].

It has been discovered that ERAS programs shorten hospital stays and are safe during major abdominal surgery in relation to anastomotic leaking. Reductions in morbidity and hospital stay are desirable because they boost bed availability and may lower the total cost of hospital stays. Additionally, there was a decrease in the usage of opioids and PONV, and an increase in pain management, all of which contributed to an earlier recovery of bowel function [27].

Beyond reducing the length of hospital stays and postoperative problems, ERAS protocols have further advantages. According to a comprehensive review, ERAS protocols have been linked to lower readmission rates, quicker bowel function recovery, and higher patient satisfaction. Additionally, it has been emphasized how important patient empowerment and education are to the perioperative care process and how they are essential to ERAS protocols [28].

The tested groups showed significant differences in pain management and bowel movement approaches. Pain scores, measured by VAS, demonstrated no significant variation between ERAS groups in the first and second cycles, yet both were notably lower than the no ERAS group in the first cycle. Drug use duration was significantly shorter in both ERAS groups across both cycles compared to the no ERAS group. Delayed intestinal motility was notably higher in the no ERAS group, reported in only five cases (22.7%), compared to the ERAS groups in both cycles.

El-Shewy et al. revealed that the ERAS group had better bowel function; the mean time for flatus passage was 1.8 days, while the mean time for a solid meal was 3.2 days [12]. Four patients (44 %) in the TC group had PONV; the first solid meal occurred 5.5 days after the first flatus passage occurred at 3.6 days. In the study by García-Botello et al., in the TC group, it took three days, whereas in the ERAS, it took one day to flatus pass [29].

There were insignificant variations in readmission rates between the groups under study and mortality. In our investigation, mortality was not mentioned. El-Shewy et al. stated that the rates of mortality and readmission did not differ statistically [12]. The only two cases that were readmitted were one in the ERAS group with wound dehiscence that was treated with tension sutures and one in the no ERAS group with abdominal collection that was emptied by ultrasound-guided aspiration. In both groups, no cases were reexamined or ended in death. This is consistent with Vlug et al. [13], with the same rates of death and readmission. Wang et al. [14] and Sarin et al. [17] revealed a lower readmission rate in the ERAS group but the death rates remained the same. Muller et al. [30] revealed a greater readmission rate in the group using ERAS [30]. It could be because of early discharge. Only in the study by Khoo et al. [15], the ERAS group experience a minor decline in death rates.

Due to the lack of random assignment in the assignment of care, residual confounding from characteristics that were not measured or that were measured could exist in our study. Additionally, even though we defined every item in the ERAS, we cannot completely eliminate any measuring inaccuracies or researcher misclassification. This is particularly true for some ERP components, such as fluid balance within the first 24 hours, which may have been absent from patient medical records and recommended more complex tests.

## Conclusions

A multidisciplinary, evidence-based program called ERAS has been connected to better patient outcomes and shorter recovery times for patients having colorectal surgery. ERAS is a workable and useful program that our hospitals can readily implement. ERAS has numerous benefits, including lower medical expenses, a considerable decrease in patient morbidity, faster recovery after surgery, shorter hospital stays, better pain management following surgery, less analgesic use, and faster bowel function.
